# Risk Factors for Dysphagia after Anterior Cervical Discectomy and Fusion with the Zero‐P implant system: A Study with Minimum of 2 Years Follow‐up

**DOI:** 10.1111/os.13170

**Published:** 2021-11-28

**Authors:** Rong Xue, Zhu‐yong Ji, Xing‐dong Cheng, Zhu‐qiu Zhang, Feng Zhang

**Affiliations:** ^1^ Department of Orthopaedics Xinghua People's Hospital Taizhou China; ^2^ Department of Orthopaedics Affiliated Hospital of Nantong University Nantong China

**Keywords:** ACDF, Cage and plate, Dysphagia, Risk factors, Zero‐P

## Abstract

**Objective:**

To evaluate the risk factors for dysphagia after anterior cervical discectomy and fusion (ACDF) with the Zero‐P Implant System by multidimensional analysis and investigated the predictive values of these risk factors for dysphagia.

**Methods:**

A retrospective analysis of 260 patients who underwent ACDF with the Zero‐P Implant System and had at least 2 year of follow‐up were performed. All patients were divided into a non‐dysphagia group and a dysphagia group. Sex, age, body mass index (BMI), intraoperative time, estimated blood loss, diabetes mellitus, hypertension, smoking, alcohol consumption, prevertebral soft‐tissue thickness, the levels of surgery, O‐C_2_ angle, C_2–7_ angle, T_1_ slope and segmental angle were analyzed. The Modified Japanese Orthopaedic Association (JOA) scoring system was used to determine functional status. NDI was used to evaluate neck pain and disability. The Bazaz grading system was chosen to evaluate dysphagia after surgery. Postoperative cerebrospinal fluid (CSF) leakage, infection, and dysphagia were recorded in both groups. An independent *t*‐test was used to compare quantitative variables, a chi‐square test was used to compare qualitative data between the two groups. To eliminate the influence of confounding factors, logistic regression was performed for multifactor regression of factors. The results were regarded as significant when the *P*‐values were less than 0.05 in this study.

**Results:**

In total, the non‐dysphagia group comprised 70 patients and the dysphagia group comprised 190 patients, with an average age of 58.33 ± 4.68 years (ranging, 42–82 years). These patients were followed up for 28.5 ± 3.5 months (range, 24–32 months). For clinical outcomes, both groups demonstrated significant improvement in the NDI and JOA scores (*P* < 0.001). According to the Bazaz dysphagia grading system, mild, moderate, and severe dysphagia were found in 50, 17, and 3 patients, respectively. In total, 37.1% (*n* = 26) had resolved by 3 month, 38.6% (*n* = 27) by 6 months, and 17.1% (*n* = 12) by 12 months. Chi‐square test results indicated that number of operated levels, operation time dT1 slope, dO‐C_2_ angle, dC_2–7_ angle, segmental angle and dPSTT were associated with a high incidence of dysphagia. Multivariate logistic regression analysis showed that number of operated levels, operation time, dC_2–7_ angle and dPSTT were significantly associated with postoperative dysphagia.

**Conclusions:**

More operated levels, more operation time, more dC_2–7_ angle and dPSTT were the risk factors for postoperative dysphagia. In additional, sufficient preoperative preparation, evaluation combined with proficient and precise surgical treatment were suggested to reduce the incidence of postoperative dysphagia when ACDF was performed.

## Graphical abstract

Risk Factors for Dysphagia after ACDF with the Zero‐P

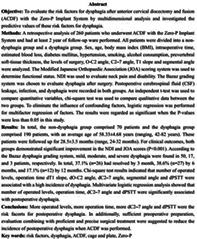

## Introduction

Cervical spondylotic myelopathy and radiculopathy are the most common spinal disorders. Surgical treatment is indicated when conservative therapy fails or when the symptoms worsen. In 1958, Cloward, Smith, and Robinson first reported that anterior cervical operation is a safe and effective method for the treatment of degenerative cervical spondylosis. Anterior cervical discectomy and fusion (ACDF) is the gold standard for the treatment of cervical spondylosis^1^, ^2^, ^3^, ^4^. Anterior cervical decompression by discectomy followed by fusion is a widely accepted and safe surgical procedure for the treatment of degenerative cervical spine disease^5^. The primary aim of this technique is to decompress the spinal cord and the affected nerve roots while restoring cervical alignment. Seventy percent to 80% of cases that were treated with complete discectomy alone led to spontaneous bony fusion; therefore, anterior cervical discectomy with or without interbody fusion is a widely accepted technique^6^. However, this technique is criticized because it does not maintain the cervical curvature, does not prevent instability and osteophyte formation, and does not preserve the vertebral disc height. Consequently, intervertebral fusion is widely recommended^7^. Initially, the iliac was often used as a substrate to achieve bone fusion. However, it may bring a considerable risk of donor site morbidity in the cervical anterior fusion technique^8^, ^9^. To prevent these adverse events, many kinds of bone graft substitutes and cages have been investigated.

Anterior cage‐plate construct is commonly used in ACDF in order to enhance segmental stability, improve cervical sagittal alignment, reduce graft extrusion and subsidence, and increase fusion rates. These techniques have their own benefits as well as potential drawbacks and adverse effects. The most often mentioned shortcomings of these techniques are the breakage or loosening of plate and screws, trachea‐esophageal injury, and neurovascular injury, have caused concerns^10^. These will accelerate disc degeneration and cause many complications, such as postoperative dysphagia for the anterior plate constructs.

Dysphagia has been considered one of the most common complications after anterior cervical spine surgery^11^, ^12^, ^13^, ^14^. Many studies have reported that an anterior plate with a lower, smoother profile may reduce the incidence of dysphagia after ACDF. The stand‐alone technique in anterior cervical interbody fusion is done by cage insertion without any additional support. It is a widely accepted and proven method, because stand‐alone cage insertion not only can help restore foraminal height but also provides immediate load‐bearing support to the anterior column and facilitates arthrodesis. To reduce these complications, the Zero‐P has been introduced and applied for ACDF. The low‐profile angle‐stable spacer Zero‐P is a new kind of cervical fusion system that is claimed to limit the potential drawbacks and complications of the above‐mentioned techniques. The device can be implanted into the intervertebral space entirely, providing adequate stability and avoiding implant contact with the prevertebral soft tissue. This device has the benefits of both of the cage and the anterior plate. A Zero‐P fusion implant in the intervertebral space after decompression will not be prominent in the vertebral column. Due to its design, Zero‐P can significantly limit the potential risks of postoperative dysphagia and degeneration of adjacent segments after the internal fixation in anterior cervical fusion surgery. Studies have shown that the thickness of the anterior plate used in anterior cervical discectomy and fusion surgery is positively correlated with the incidence rate of postoperative dysphagia and its complications^15^. Therefore, reducing the thickness of the anterior plate could considerably reduce the rate of the incidence of postoperative dysphagia and its complications.

However, there is still considerable controversy about the stand‐alone cage technique because of complications, such as, anterior cage migration, lower immediate stability with the cage, segmental kyphosis, and cage subsidence. Maintenance of the cervical curvature and disc height, prevention of cage subsidence, and conferring greater stability to the operated segment are often mentioned as reasons for the implantation of an anterior plating system. In addition, it can prevent the development of kyphosis and increase the bone fusion rate^16^, ^17^, ^18^. Thus, the fusion procedure with a cage and a plate seemed to be the gold standard in the treatment of patients with symptomatic cervical spondylosis. Nevertheless, plate migration, acceleration of disease of the adjacent segment, and dysphagia are the most frequently mentioned drawbacks associated with the anterior cage and plate technique^19^, ^20^. Studies have shown that the Zero‐P implant could demonstrate similar biomechanical properties as the traditional anterior cervical cage and plate, such as allowing good activity and stability of the surgical segments^21^. The group of Scholz *et al*. also selected 38 patients with cervical spondylosis who underwent Zero‐P cervical interbody fusion surgery for an average of 8 months follow‐up. The follow‐up results showed that all patients obtained satisfactory bone fusion and functional recovery^22^. In a meta‐review, the results suggest that the new Zero‐P internal fixation system could lead to a good fusion rate similar to that of the traditional cervical fusion surgery with a cage and anterior plate. For intraoperative time and intraoperative blood loss, it was difficult to conclude which is the better technique in terms of operation trauma.

Although the risk factors for postoperative dysphagia are multidimensional, the literature lacks a comprehensive analysis of them. The purpose of this study was to: (i) assess the incidence of dysphagia after anterior cervical spine surgery with the Zero‐P implant system; (ii) investigate the predictive values of the risk factors for dysphagia; and (iii) assess the correlation between cervical sagittal balance and dysphagia.

## Materials and Methods

### General Information

A retrospective analysis was performed in our hospital from January 2014 to July 2017. This retrospective study was approved by the Ethics Committee of our Hospital. All of the patients were recruited after providing informed consent for analysis of their clinical data. Radiologic diagnoses were established in each patient through routine preoperative cervical anteroposterior, lateral, flexion‐extension radiographs and cervical magnetic resonance imaging or computed tomography scans. All patients had symptoms and signs of neural compression that were refractory to conservative treatment. All patients underwent ACDF with Zero‐P. Patients were divided into the postoperative dysphagia group and the non‐dysphagia group. Sex, age, BMI, diabetes, hypertension, smoking, alcohol consumption, duration of symptom, surgery blood loss, degree of prevertebral soft tissue swelling, and O‐C_2_ angle, C_2‐7_ angle, T_1_ slope, the operative segment and the number of operative segments were collected.

### Inclusion and Exclusion Criteria

Inclusion criteria was as follows: (i) patients were diagnosed as cervical spondylotic myelopathy or cervical spondylotic radiculopathy; (ii) the clinical symptoms and signs of the disease were consistent with the results of imaging examination; (iii) conservative treatment was not effective for more than 6 months; (iv) ACDF is suitable for the segment, the fused segment prosthesis was Zero‐P; (v) preoperative swallowing was normal; and (vi) the follow‐up time was at least 2 years.

Exclusion criteria was as follows: (i) preoperative dysphagia; (ii) a history of other disorders that may trigger symptoms of dysphagia, such as craniocerebral trauma or cerebral infarction; (iii) received revision surgery; and (iv) c ombined with cervical deformity, tumor, severe osteoporosis, ankylosing spondylitis and rheumatoid arthritis;

### Surgical Procedure

All the surgical procedures were performed by the same senior surgeon using the standard Smith‐Robinson approach. The cartilaginous disc endplate was carefully removed and care was taken to avoid excessive damage to the bony endplate. The posterior osteophytes were removed by curettes and Kerrison rongeurs. After complete decompression of the spine cord and nerve roots, the ideal sizes of cages were selected by radiographic assisted trials. The width of the cage was determined by the distance between the two Luschka's joints, and the height of the cage was determined by different trails under radiography, when trails are tightly fitted in the disc space without over‐distraction of the disc space or facet joints. Porous bioceramic artificial bone was used to fill in the cage in all patients. Proper sized devices (Zero‐P, USA) or cages along with anterior cervical plate were inserted and the anchorage systems were inserted in the vertebral bodies under the fluoroscopic guidance.

Postoperatively, all patients received prophylactic anti‐infective therapy for 2 days. Methylprednisolone was administered intravenously for 3 days, with doses being 40, 20, and 10 mg. Drainage fluid amount and characteristics were monitored. The drainage tube was removed within 24 to 48 h postoperatively. All patients were treated with cervical collar fixation for 3 months.

### Evaluation Criteria

The time of operation and intraoperative bleeding volume were calculated by two residents. In addition, intraoperative complications such as dural tear was recorded.

All patients were followed up for at least 24 months after operation. Data were collected preoperatively and at 3, 6, 12, 24 months and final follow‐up after surgery.

### Clinical Outcome

#### Modified Japanese Orthopaedic Association (JOA) Score and Recovery Rate

The Modified Japanese Orthopaedic Association (JOA) scoring system was used to determine functional status before surgery and the final follow‐up visit. The recovery rate (%) at the final follow‐up visit was calculated by using the Hirabayashi method: (postoperative JOA score—preoperative score)/(17 preoperative score) × 100%. And the recovery rates were graded as follows: 75% and greater, excellent; 50% to 74%, good; 25% to 49%, fair; and less than 25%, poor.

#### Neck Disability Index (NDI)

NDI was used to evaluate neck pain and disability; NDI was assessed at preoperative and at last follow‐up. NDI contains 10 self‐reported items, including: pain intensity, personal care, lifting, reading, headache, concentration, working, sleeping, driving, and entertainment. Each item is scored from zero to five. The final score was presented as the percentage of the maximal score. Final NDI score is calculated as (total score/(5 × number of questions answered)) × 100%. Zero percent to 20% is considered mild dysfunction, 21% to 40% is moderate dysfunction, 41% to 60% is severe dysfunction, and 61% to 80% is considered as disability. 81% to 100% is to either long‐term bedridden or exaggerating the impact of pain on their life.

#### Dysphagia Grade

The Bazaz grading system was chosen to evaluate dysphagia after surgery. The scores of the Bazaz grading system were ranked as follows: 0 none, 1 mild, 2 moderate and 3 severe, representing no episodes of swallowing problems, rare episodes of dysphagia, occasional swallowing difficulties with specific foods and frequent swallowing difficulties with most foods, respectively^23^.

### Radiographic Assessment

#### C_2_ –C_7_ Lordosis

The C_2_–C_7_ lordotic angle was defined as the Cobb angle formed by the inferior endplate of C_2_ and C_7_ on a standing lateral radiograph. C_2_–C_7_ lordotic angle was measured on the lateral radiograph before and after surgery.

#### Segmental Lordosis

The segmental lordotic angle was defined as the Cobb angle formed by the upper endplate of the uppermost body and the lower endplate of the lowermost body at the operated segment.

#### T_1_ Slope

The T_1_ slope angle was formed by the T_1_ upper endplate and the horizontal reference line.

#### O‐C_2_ Angle

The O‐C_2_ angle was formed by the McGregor's line and the inferior margin of the C_2_ vertebrae.

#### Prevertebral Soft Tissue Thickness

The prevertebral soft tissue thickness (PSTT) was measured at the extended line of the anteroposterior diameter which ends at the posterior aspect of the trachea from C_2_ to C_7_, and the anteroposterior diameter was measured between the centers of the posterior and anterior cortex of each vertebral body^24^, ^25^.

Furthermore, the differences of parameters before and after operation were analyzed (dC_2–7_ angle, dSL, dO‐C_2_ angle, dT_1_ slope and dPSTT).

### Complications

Postoperative cerebrospinal fluid (CSF) leakage, infection, and dysphagia were recorded in both groups.

### Statistical Analysis

All statistical analyses were performed with the statistical program SPSS version 22.0 (SPSS Inc., Chicago, IL, USA). Continuous variables were expressed as mean ± standard deviation. A paired *t*‐test was used to analyze changes between pre‐ and postoperative parameters. An independent *t*‐test was used to compare quantitative variables between the two groups. A chi‐square test was used to compare qualitative data between the two groups. To eliminate the influence of confounding factors, ordinal logistic regression was performed for multifactor regression of factors with a *P* value less than 0.05 in the single factor analysis. The results were regarded as significant when the *P*‐values were less than 0.05 in this study.

## Results

### General Results

**Fig. 1:**
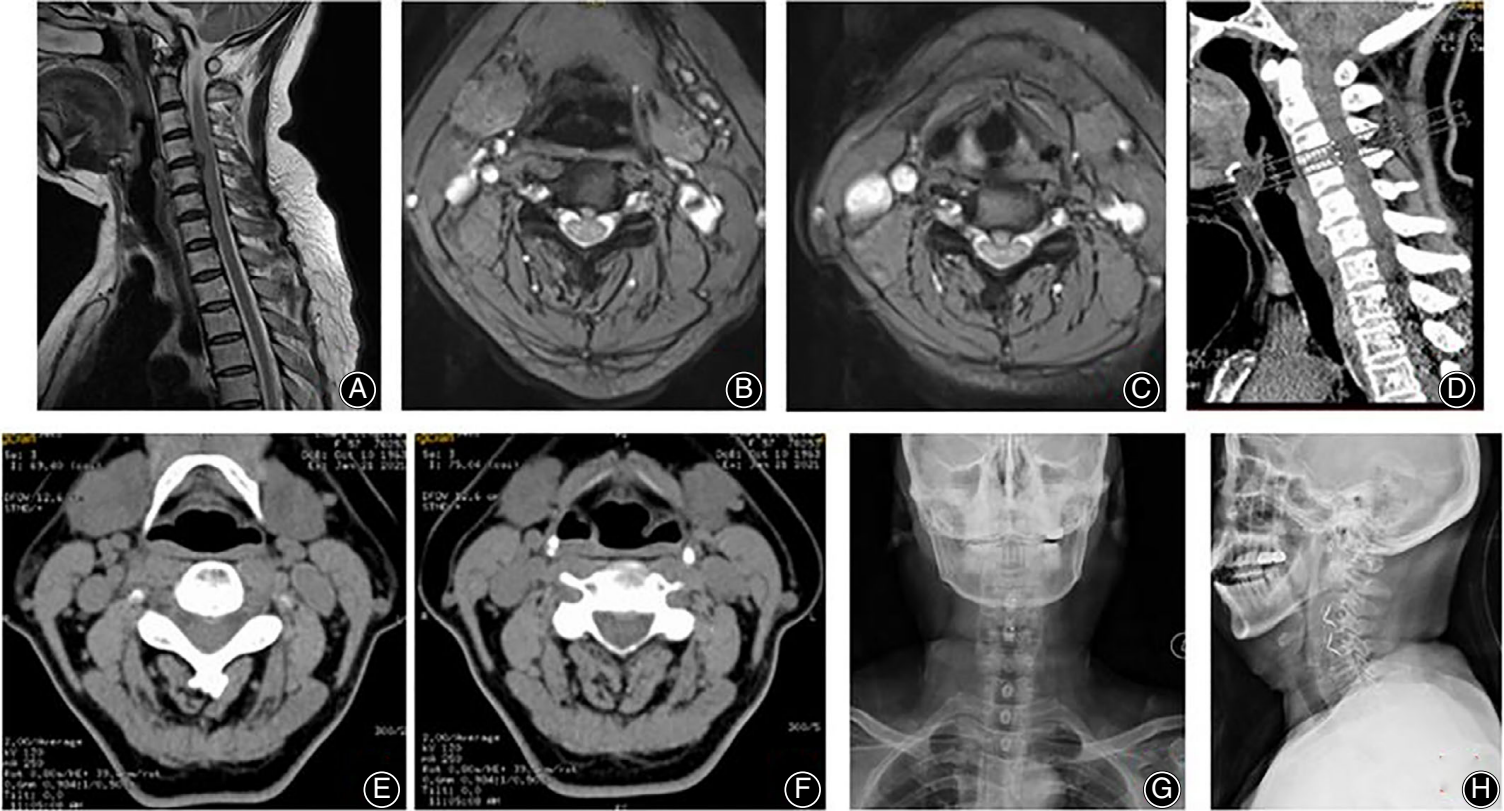
A 57‐years female patient with cervical spondylosis myelopathy. The patient had mild dysphagia after surgery, and the symptoms disappeared 3 months after surgery. (A–C) MRI showed herniated discs at C_3/4_ and C_4/5_ with spinal cord compression. (D–F) CT showed no calcification of OPLL or intervertebral disc. (G–H) X‐ray showed a satisfactory fixation position at 2 years of follow‐up.

In total, 260 consecutive patients were enrolled in this study, including 109 males and 151 females with an average age of 58.33 ± 4.68 years (ranging, 42–82 years). These patients were followed up for 28.5 ± 3.5 months (range, 24–32 months). The diagnoses were cervical spondylotic myelopathy in 105 cases, cervical spondylotic radiculopathy in 75 cases, mixed cervical spondylosis in 34 cases, and posterior longitudinal ligament ossification in 46 cases. Figure 1 shows a typical case.

A total of 70 patients developed dysphagia after surgery. The main symptoms included difficulty in swallowing or an inability to swallow when swallowing liquids; food retention in the throat fatigue during swallowing or an inability to swallow solids and pain or a burning sensation with swallowing. According to the Bazaz dysphagia grading system, mild, moderate, and severe dysphagia were found in 50, 17, and three patients, respectively. In total, 37.1% (*n* = 26) had resolved by 3 months, 38.6% (*n* = 27) by 6 months, and 17.1% (*n* = 12) by 12 months. Five patients underwent esophageal angiography, with no significant esophageal stenosis or esophageal fistula observed, and four patients underwent electronic fiber laryngoscopy, which showed no significant laryngeal mucosal injury, vocal cord relaxation, or paralysis. At final review, dysphagia was still present in four patients (5.8%), including three patients with mild dysphagia and one patient with moderate dysphagia (Table 1).

**TABLE 1:** Summary of dysphagia and dysphonia data

Postoperation	*n*	Follow‐up (3m/6m/12m/24m)		
		Mild	Moderate	Severe
Mild	50	24/9/10	0/0/0/0	0/0/0/0
Moderate	17	7/3/1/1	10/3/1/1	0/0/0/0
Severe	3	0/0/1/1	0/1/1/1	3/1/0/0
Total	70	31/12/3/2	10/4/2/2	3/1/0/0/0

### Clinical Characteristics

The dysphagia group consisted of 70 patients while the non‐dysphagia group consisted of 190 1patients. For clinical outcomes, both groups demonstrated significant improvement in the NDI and JOA scores (*P* < 0.001). We used univariate analysis to evaluate the independent factors, including sex, age, duration of symptom, BMI, operation time, blood loss, smoking, operated levels, alcohol abuse and BMI to see which were related to postoperative dysphagia. Chi‐square test results indicated that number of operated levels (*P* < 0.001) and operation time (*P* = 0.012) were associated with a high incidence of dysphagia(Table 2).

**TABLE 2:** Demographic and clinical characteristics

Item	Dysphagia group	Non‐dysphagia group	*t* value	*P* value
Patients	70	190	0.010	0.312
Age (years)	59.2 ± 8.2	57.7 ± 5.9	9.431	0.456
Gender: male	34	75	0.032	0.394
Duration of symptoms (m)	20.9 ± 3.6	22.1 ± 3.2	1.341	0.221
Smoke	12	29	3.512	0.654
Alcohol Abuse	11	32	0.345	0.481
Follow‐up (m)	29.7 ± 3.7	27.1 ± 5.5	2.459	0.278
Number of operated levels	3.2 ± 0.7	1.4 ± 0.5*	3.451	<0.001
Operation time (min)	109.4 ± 20.3	73.2 ± 15.6*	0.312	0.012
Blood loss (ml)	60.1 ± 22.2	40.9 ± 18.3	1.742	0.342
Pre‐OP NDI	2.02 ± 1.41	2.19 ± 1.54	3.401	0.348
Prost‐OP NDI	1.89 ± 2.16	2.01 ± 1.73	0.321	0.299
Pre‐OP JOA	10.5 ± 1.1	10.7 ± 1.5	0.491	0.078
Post‐OP JOA	15.7 ± 1.5	15.8 ± 1.2	2.983	0.181
Recover ratio of JOA (%)	72.6 ± 16.1	71.1 ± 23.1	3.912	0.230
Outcome (excellent/good, %)	100	100	1.000	1.000

* There was a statistically significant difference between two group, *P* value < 0.05.

### Imaging Parameters

Furthermore, imaging parameters including dO‐C_2_ angle, dC_2–7_ angle, dT_1_ slope, segmental angle and dPSTT were also evaluated. The result indicated that dT_1_ slope, dO‐C_2_ angle, dC_2–7_ angle, segmental angle and dPSTT were related to postoperative dysphagia (*P* < 0.005) (Table 3).

**TABLE 3:** Imaging parameters between the 2 groups

Item	Dysphagia group	Non‐dysphagia group	*t* value	*P* value
Pre‐OP O‐C_2_ angle (°)	16.2 ± 6.4	16.5 ± 4.5	4.261	0.123
Prost‐OP O‐C_2_ angle (°)	14.2 ± 3.1	14.3 ± 3.2	5.332	0.061
Pre‐OP C_2–7_ angle (°)	15.1 ± 8.6	15.4 ± 7.9	2.081	0.235
Post‐OP C_2–7_ angle (°)	16.3 ± 6.1	16.1 ± 6.2	0.154	0.431
Pre‐OP T_1_ slope (°)	26.2 ± 4.7	26.4 ± 4.7	2.541	0.382
Post‐OP T_1_ slope (°)	30.4 ± 6.7	26.4 ± 6.7*	2.649	<0.001
Pre‐OP segmental angle (°)	7.5 ± 6.9	7.5 ± 6.3	1.509	0.512
Post‐OP segmental angle (°)	14.5 ± 6.2	8.2 ± 5.9*	0.639	0.021
Pre‐OP PSTT (mm)	9.2 ± 1.5	9.3 ± 1.4	0.134	0.652
Pre‐OP PSTT (mm)	13. 6 ± 3. 7	10.9 ± 1.0*	2.124	0.218

* There was a statistically significant difference between two group, *P* value < 0.05.

### Multivariate Logistic Regression Analysis

Multivariate logistic regression analysis showed that number of operated levels (*P* = 0.037), operation time (*P* < 0.001), dC_2–7_ angle (*P* = 0.022) and dPSTT (*P* < 0.001) were significantly associated with postoperative dysphagia. However, dT1 slope, dO‐C_2_ angle and slaws not significantly associated with postoperative dysphagia in the multivariate model (Table 4).

**TABLE 4:** The results of the logistic regression analysis between related factors and dysphagia

Clinical factors	Odds ratio	95% *CI*	*P*
Number of operated levels	1.917	1.0–3.3	0.037
Operation time	2.46	2.0–5.6	<0.001
dO‐C_2_ angle	0.123	1.9–7.3	0.022
dC_2–7_ angle	2.323	0.6–3.1	0.041
dT_1_ slope	2.113	1.9–4.9	0.215
Segmental angle	3.211	1.0–3.3	0.169
dPSTT	2.063	3.3–8.3	<0.001

## Discussion

### Risk Factors of Dysphagia

Dysphagia contributes to higher self‐reported disability and lower physical health status. The most probable explanation for postoperative dysphagia is that it is a multifactorial phenomenon, explained by esophageal retraction, direct cervical plate stimulating the esophagus, prevertebral swelling, among others. However, the pathophysiology and risk factors of postoperative dysphagia are not fully understood. Persistent and severe dysphagia may lead to various degrees of discomfort, and increase the risk of various complications, such as difficulty in eating or drinking, bronchospasm, aspiration pneumonia, dehydration, asphyxia and malnutrition.

The incidence of dysphagia after anterior cervical spine surgery has been reported as ranging from 1% to 80%. Baron *et al*. believed that the incidence of transient dysphagia after anterior cervical fusion was as high as 80% and that the symptoms of most patients were relieved after treatment^26^. These greatly varying incidence rates may be related to factors such as surgical approach, sample size, and differences in case selection and evaluation methods, particularly regarding the criteria used to define postoperative dysphagia^27^, ^28^. Theoretically, if an anterior cervical titanium plate is placed directly into the esophagus, the titanium plate may affect the incidence of postoperative dysphagia. Any mechanical irritation or impingement of the esophagus may cause symptoms of dysphagia. No‐notch implants were considered to be associated with anterior plate+cage. Our results show that the incidence of dysphagia of patients used Zero‐P system at 2 years follow‐up (1.48%) was lower than that of the previous reported.

Many scholars have developed classification systems to define and classify postoperative dysphagia, but the inconsistent usage and lack of consensus limit its research progress. X‐ray fluoroscopic examination of swallowing function has long been regarded as the gold standard for assessing swallowing difficulty. The Bazaz grading system is widely used to assess the incidence of dysphagia after cervical spine surgery. But it has many shortcomings. This scale is based on qualitative information collected by an investigator to assess the patient's subjective sensation of difficulty when swallowing liquids and solids, possible sensory disruptions causing postoperative dysphagia may be challenging to explain and may not reflect accurate clinical outcomes. In addition, the correlation between the indicators of dysphagia and subjective performance is not clear. Anderson believed that the self‐assessment questionnaire for patients' main symptoms might be a relatively reliable method for evaluating clinical dysphagia after anterior cervical surgery^29^. The SWAL QOL score was recommended to be used to assess the degree of dysphagia. Postoperative dysphagia was evaluated using the Bazaz scoring system in our results, and it was found that dysphagia was a common early complication after anterior cervical surgery, and the incidence and severity of dysphagia gradually decreased over time. After 1 year of follow‐up, almost all patients had resolved their dysphagia, and only a few still had mild dysphagia. Bazaz's prospective study of 249 patients who underwent anterior cervical surgery found that the incidence of dysphagia was 50.2%, 32.2%, 17.8%, and 12.5% at 1, 2, 6, and 12 months after surgery, respectively. At 6 months after surgery, only 4.8% of patients had moderate or severe dysphagia, which was basically consistent with the results of this study.

### Correlation between Cervical Sagittal Balance and Dysphagia

Recently, several studies have compared the clinical outcomes spacer and cervical sagittal balance in ACDF for treating cervical disorders^30^, ^31^, ^32^. And the result of some studies indicated the Cobb angle, T_1_ slope and other parameters are closely related to clinical outcomes^33^, ^34^, ^35^. To the best of our knowledge, few reports have described the effect of the difference between postoperative and preoperative sagittal balance on postoperative dysphagia after ACDF with Zero‐P. Consequently, one objective of this retrospective analysis was to summarize and identify the effect of the cervical sagittal balance and other possible related factors on dysphagia. And provides for future spinal surgeries with evidence on how to reduce the incidence of dysphagia after ACDF with Zero‐P.

Izeki believed that posterior cervical surgery, such as posterior occipito‐cervical fusion, if the surgery changes the physiological curvature, will result in mechanical strictures of oropharynx, which may lead to postoperative swallowing disorders^36^. This provides an idea for us to study the swallowing disorder caused by the overall curvature change after cervical spine surgery. Our study indicated that dO‐C_2_ angle and dC_2–7_ angle were significantly related with postoperative dysphagia.

Khaki *et al*. believed that anterior cervical surgery could affect the throat stage in the four stages of swallowing process, thus leading to postoperative dysphagia^37^. Based on the previous studies, we concluded that the posterior pharyngeal wall protruded forward due to the large angle of dC_2‐7_ angle after surgery, which reduced the throat volume and affected the squeezing effect of pharynx during eating, thus leading to dysphagia. However, there is still a lack of direct imaging evidence to confirm this view, and the hypothesis proposed by us can only explain to a certain extent the mechanism of the dC_2‐7_ angle and postoperative dysphagia. In the anterior cervical decompression, the intervertebral space should be extended as far as possible with the use of a retractor to restore the normal curvature of the cervical spine and reduce postoperative cervical degeneration. The results of this study suggest that excessive expansion of cervical spine space may cause excessive changes in C_2‐7_ Angle, which may lead to forward protrusion of the posterior pharyngeal wall, resulting in postoperative dysphagia. Therefore, the recovery of anatomical force line should not be pursued only, but also the change of cervical curvature should be controlled to reduce the occurrence of postoperative dysphagia in ACDF. This suggests that intraoperative control of C_2_–C_7_ angle within a reasonable range may reduce the probability of postoperative dysphagia symptoms.

Operative time was another factor associated with dysphagia in our study. In a prospective study of 38 patients undergoing single‐segment and double‐segment ACDF surgery, found that extended duration of surgery was the only variable associated with the severity of dysphagia 12 weeks after surgery^38^. We hypothesized that prolonged traction of the trachea and esophagus would inevitably lead to more severe soft tissue swelling. Therefore, in complex cervical spine surgery, where the operation is expected to take a long time, surgery by a senior cervical surgeon may reduce the incidence of postoperative dysphagia more than surgery by a junior physician or resident.

### Limitations

There are some limitations in the current study. First, the sample size of the retrospective study was small, though it was adequate to evaluate the variables. A further prospective study should be designed using a standardized scoring system and postoperative examination. Second, the follow‐up period was relatively short, with a mean of 28.5 months. Therefore, the long‐term clinical effect should be evaluated. Third, not all potential risk factors, such as the thickness and length of the plate, were considered in the statistical analysis. In addition, the mechanisms by which the T_1_ slope affects the development of dysphagia after ACDF with the Zero‐P implant System are not completely clear. Therefore, multicenter and randomized controlled studies are needed to verify our conclusions in the future.

## Conclusion

Dysphagia is one of the most common complications after anterior cervical spine surgery. Multivariate logistic regression analysis showed that number of operated levels, operation time, dC_2‐7_ angle and dPSTT were significantly associated with postoperative dysphagia. In additionally, sufficient preoperative preparation, evaluation combining with proficient and precise treatment measures are suggested to reduce the incidence of postoperative dysphagia when ACDF is performed.

## Availability of Data and Materials

The datasets generated during and/or analyzed during the current study are publicly available. The datasets generated during and/or analyzed during the current study are available from the corresponding author on reasonable request.

## Authors’ Contributions

X.R. conducted the trials and drafting the manuscript. J.Z.Y. and C.Y.Q. participated in the design of the study and performed the trial. Z.Z.Q. and C.X.D. supervised and coordinated the study. All authors read and approved the final manuscript. Z.F. is thecorresponding author of this manuscript.

## Ethics Approval

This retrospective study was approved by the Ethics Committee of our Hospital. All of the patients were recruited after providing informed consent for analysis of their clinical data.
